# Tick-Borne Co-Infection in Lyme Disease: Clinical Impact, Diagnostic Challenges, and Therapeutic Perspectives

**DOI:** 10.3390/microorganisms14020325

**Published:** 2026-01-30

**Authors:** Georgi Popov, Dzhaner Bashchobanov, Radina Andonova

**Affiliations:** Clinic of Infectious Diseases, Sofiamed Hospital, 1797 Sofia, Bulgaria; popovg@abv.bg (G.P.); radina6@mail.bg (R.A.)

**Keywords:** Lyme disease, co-infection, ticks, diagnostic, treatment, clinical presentation, *Babesia* spp., *Anaplasma phagocytophilum*, *Ehrlichia* spp., *Borrelia miyamotoi*

## Abstract

Tick-borne co-infections are an increasingly recognized and clinically important aspect of Lyme borreliosis, particularly in regions where *Ixodes* ticks transmit a wide range of bacterial, protozoan, and viral pathogens. In addition to *Borrelia burgdorferi sensu lato*, these ticks frequently harbor microorganisms such as *Babesia* spp., *Anaplasma phagocytophilum*, *Ehrlichia* spp., *Borrelia miyamotoi*, *Bartonella* spp., and several tick-borne viruses. Co-infections may increase disease severity, prolong symptom duration, and contribute to atypical or overlapping clinical presentations, thereby complicating diagnosis and management. Growing evidence from epidemiological studies, clinical case series, and experimental in vivo and in vitro models indicates that pathogen–pathogen and pathogen–host interactions can modulate immune responses and influence disease progression. Diagnostic challenges arise from non-specific clinical features and limitations of current laboratory methods. From a therapeutic perspective, although standard antibiotic regimens for Lyme disease are effective against some bacterial co-infections, they do not provide coverage for protozoan or viral agents, necessitating pathogen-specific and, in some cases, combination treatment strategies. This review synthesizes current knowledge on the epidemiology, clinical impact, diagnostic limitations, and treatment approaches for tick-borne co-infections associated with Lyme disease, and highlights critical evidence gaps and future research directions to improve patient outcomes.

## 1. Introduction

Lyme disease (LD) is a multisystemic illness and the most common tick-borne disease in the Northern Hemisphere. It is caused by spirochetes of the *Borrelia burgdorferi sensu lato complex* (*Bbslc*) and is transmitted to humans through bites from black-legged ticks of the genus *Ixodes* [1,2]. In Europe, the main causative agents are *Bo. burgdorferi sensu stricto*, *Bo. garinii*, *Bo. afzelii*, *Bo. bavariensis*, and the recently identified *Bo. mayonii*; their primary vectors are *I. ricinus* and *I. persulcatus* [1,3]. In Asia, *Bo. garinii* is the predominant causative agent, transmitted by *I. ricinus*, *I. persulcatus*, and *I. ovatus* [4]. In North America, *B. burgdorferi sensu stricto* and *Bo. mayonii* are the most common cause, with *I. scapularis* and *I. pacificus* serving as principal vectors [1,5,6].

*Ixodes* ticks are a major public health concern because they are widespread, increasingly found near humans, and can carry multiple pathogens at once [7,8,9,10]. Besides *Bbslc*, they can spread bacteria like *Borrelia miyamotoi* (*Bo. miyamotoi*)-relapsing fever borreliosis [7,11] and *Anaplasma phagocytophilum* (*An. phagocytophilum*)-human granulocytic anaplasmosis [7,12], as well as *Neoehrlichia mikurensis* (*N. mikurensis*)-Neoehrlichiosis and *Ehrlichia ssp.*-human ehrlichiosis [7,13,14]. Transmissible viruses include *tick-borne encephalitis virus* (*TBEV*), *Powassan virus* (*POWV*), *Louping Ill Virus*, and *Eyach virus* (*EYAV*) [7,15,16,17,18]. *Ixodes* ticks also transmit parasites such as *Babesia divergens* (*Ba. divergens*), *Ba. microti*, *Ba. duncani*, and *Ba. venatorum*, which causes human babesiosis [7,19,20].

The potential for ticks to transmit multiple pathogens highlights the growing concern of co-infections associated with Lyme disease. Simultaneous infection with several pathogens can alter clinical presentations, complicating both the diagnosis and management of these diseases [21]. This literature review aims to explore the link between the presence of diverse tick-borne pathogens and the increased complexity in diagnosing and treating Lyme disease, with particular attention to selecting appropriate therapeutic strategies for managing co-infections.

## 2. Epidemiology of Vectors and Co-Occurrence Pathogens

As mentioned above, different species of *Ixodes* ticks are found across various continents and regions, each transmitting distinct species of *Bbslc*. Additionally, these ticks are associated with different co-infections.

**Figure 1:**
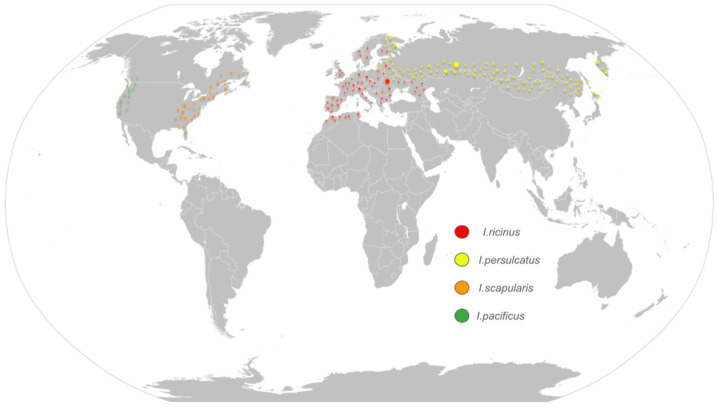
Worldwide distribution of *Ixodes* ticks.

*I. ricinus*, which is the main vector of Lyme disease in Europe (*Bo. burgdorferi sensu stricto* (*Bbss*), *Bo. garinii*, *Bo. afzelii*, *Bo. bavariensis*), can also transmit *TBEV*, *EYAV*, *Anaplasma phagocytophilum*, *Neoehrlichia mikurensis*, *Ba. divergens*, *Ba. venatorum*, and various species of *Rickettsia* [7]. Numerous European studies (Switzerland [22,23], Poland [24,25], Scandinavian countries [26], Bulgaria [27], etc.) demonstrate that *I. ricinus* can be infected with more than one pathogen, with the most common combinations being between *Bbsl* and *An. phagocytophilum* or *Babesia* ssp. [28], which directly increase the risk of co-infections in humans (Table 1 and Figure 1).

*I. persulcatus* is the main vector of LD in Northern Europe, Russia, and Asia. In addition to *Bbslc*, they can also transmit *TBEV*, *Omsk hemorrhagic fever virus* (*OHFV*), and *Bo. miyamotoi*, *An. phagocytophilum*, *Neoehrlichia mikurensis*, *Ehrlichia muris* (*E.muris*), *Riketsia* spp., and *Ba. venatorum* [7]. A study in Mongolia found that 49% of a total of 346 *I. persulcatus* ticks tested were infected with *Bbslc*, *Ehrlichia* sp.—16%, *A. phagocytophilum*—13.5%, *Bo. mayomatoi*—4.9% and *TBEV*—1.7%. Co-infections between Bbslc and the other pathogens studied were as follows (presented as a percentage of ticks with Bbslc and another pathogen—55 ticks)—*TBEV*—3.6%, *Bo. mayomatoi*—10.9%, *A. phagocytophilum*—30.9%, *Ehrlichia* sp.—36.4%. In 18.2% (10 ticks) of the ticks infected with *Bbslc*, in which more than one pathogen was detected, triple infections were found—in one of them a combination of *TBEV*, *Bbslc*, and *A. phagocytophilum*, and another had the following combination: *TBEV*, *Bbslc*, and *Ehrlichia* sp. The remaining eight were found to be simultaneously infected with *Bbslc*, *Ehrlichia* sp., and *A. phagocytophilum* [30]. Russia provides some of the best-documented data on co-infections in *I. persulcatus*, a tick species endemic to northern and eastern Eurasia. Studies from various regions—including Saint Petersburg [31], Vologda [32], Kirov [33], and Karelia [34]—show a high frequency of multiple pathogens within individual ticks. The prevalence of co-infections varies between these geographical areas. In the study by Alekseev et al. [31] from the St. Petersburg region, a total of 1282 adult ticks and 19 nymphs were collected. Among these, 120 ticks harbored two pathogens, most commonly a combination of *Bo. afzelii* and *Bo. garinii* (43%), followed by *Bo. garinii* and *Bbss* (15.5%), and *Bo. afzelii* and *Ehrlichia muris* (15.5%). Triple infections were found in 22 ticks, most frequently involving *Bo. afzelii*, *Bo. garinii*, and either *E. muris* (22.7%) or *TBEV* (22.7%). Additionally, *Ba. microti* and *A. phagocytophilum* were detected. In Eremeeva et al. [32]’s study from the Vologda region, populations of *I.persulcatus.* ticks were examined over two consecutive years for *Bbslc*, *An. phagocytophilum*, *E. muris*, and certain *Rickettsia* species. The most common in the presence of co-infections were *Bbslc* with *An. phagocytophilum* and *Bbslc* with *E. muris*. In the Kirov region study [33], 322 ticks were examined; 155 were infected with at least one pathogen. Out of these, a quarter contained more than one pathogen (36 with double infections and 5 with triple infections). In the Karelia region [34], ticks collected between 2007 and 2018 were studied. No *An. phagocytophilum* carriage was detected. Of 400 *I. persulcatus* ticks, 142 harbored mixed infections, most commonly *Bbslc* with *TBEV* or *Bbslc* with *Ehrlichia* spp. A study from Finland [35] examined 2014 *I. ricinus* and 1451 *I. persulcatus* ticks. Of the *I. ricinus* ticks, 49 (2.4%) were found to carry more than one pathogen, most commonly *Bbslc* and *Rickettsia* spp. (1.5%). Among *I. persulcatus* ticks, only 11 (0.8%) carried multiple pathogens, with *Bbslc* and *Rickettsia* spp. present in each case.

*I. scapularis* is one of the primary vectors of LD in North America. In addition to *Bbslc*, it can transmit other pathogens, including bacteria (*An. phagocytophilum*, *E. muris*, *Bo. mayonii*, etc.), viruses (*POWV*, *South Bay virus*, etc.), and eukaryotes/parasites (*Ba. microti* and *Ba. duncani*) [7]. Numerous studies [10,36,37] have investigated the range of pathogens found in these ticks. In one study conducted in Wisconsin [36], 112 ticks were examined: 42.9% carried *Bbslc*, 46.4% carried *Rickettsia* spp., 10.7% *A. phagocytophilum*, 3.6% *Ehrlichia* spp., 0.9% *Bo. mayonii*, 1.8% *POWV*, 51.8% *South Bay virus*, and 8.9% *Ba. microti*. A significant positive correlation was found between infection with *South Bay virus* and *Bb* (*p*-value = 1.4 × 10^−3^). In another study from Minnesota [37], 1240 nymphs of the species *I. scapularis* were examined, with 25.24% found to be infected with *Bbss*. Additionally, *Bo. mayonii*, *Bo. miyamotoi*, *An. phagocytophilum*, *E. muris*, *Ba. microti*, and *POWV* were detected. Co-infections occurred in 7.26% of the nymphs, involving combinations of two, three, or even four pathogens. The most common co-infections were between *Bbss* and *An. phagocytophilum* or *Bbss* and *Ba. microti*. The most frequent triple co-infection involved *Bbss, An. phagocytophilum*, and *Ba. microti*, though combinations with all studied pathogens were found. Notably, *Bbss* was always present in nymphs harboring four pathogens. A study from New Jersey [10] examined 662 adults and nymphs of the species *I. scapularis* collected between 2020 and 2021. One quarter of the nymphs and slightly more than half of the adults were carriers of pathogens. The most commonly detected pathogen was *Bbslc*. The study also reported data on specimens carrying two or three pathogens, with the most frequent co-infection combinations being *Bbslc* with *An. phagocytophilum*, and *Bbslc* with *Ba. microti*. The most common triple infection involved *Bbslc*, *An. phagocytophilum*, and *POWV*.

*I. pacificus* is another known vector of Lyme disease (LD) in North America, besides *Bbslc*. It can also transmit other pathogens, including *Bo. miyamotoi*, *A. phagocytophilum*, and *Ba. duncani* [7]. A Canadian study [38] examined 9858 *I. scapularis* and 691 *I. pacificus* ticks, finding that *Bb* was the most common pathogen: 18.8% in *I. scapularis* and 0.3% in *I. pacificus*. No other pathogens or co-infections with *Bb* were found in *I. pacificus*. In *I. scapularis*, the most frequent combination was *Bb* with *A. phagocytophilum*. A large-scale US study [39] analyzed 13,400 *Ixodes* ticks (*I. scapularis* and *I. pacificus*) from 17 states and the District of Columbia: 12,636 were *I. scapularis* and 764 were *I. pacificus*. All were tested for *Bbss*, *Bo. miyamotoi*, *Bo. mayonii*, *A. phagocytophilum*, and *Ba. microti*. *Bbss* was the most frequently isolated pathogen (26.7%), mainly in *I. scapularis*, while only 2.35% of *I. pacificus* carried it. This trend—lower rates of pathogen detection in *I. pacificus* compared to *I. scapularis*—was also seen for the other three microorganisms studied. The most common co-infection was observed between *Bbss* and *A. phagocytophilum* (2.2%). In *I. pacificus* ticks, however, none of the three pathogen combinations studied (*Bbss* + *A. phagocytophilum*, *Ba. microti*, or *A. phagocytophilum* + *Ba. microti*) were detected.

## 3. The Clinical Characteristics of Co-Infections in Lyme Disease

Ticks can carry more than two pathogens. Studies from Europe and North America have shown that more than one pathogen can be isolated from a single tick [22,23,24,25,26,27,28,30,31,32,33,34,35,36,37,38,39]. Experimental data confirm that *Ixodes* ticks can transmit at least two pathogens with one bite [40], most commonly *Borrelia burgdorferi* together with *Anaplasma phagocytophilum* or *Babesia microti* [20,21,22,23,24,25,26,27,28,29,30,31,32,33,34,35,36]. However, there are no well-documented human cases in the literature in which three or more pathogens have been transmitted by a single tick bite. This may be due to several biological factors, including the specific location of each pathogen within the tick, interactions between co-infecting pathogens, and their clearance by the human immune system [41]. Thus, while multi-pathogen transmission from a single bite is possible, it appears to be relatively uncommon.

### 3.1. Co-Infection with Borrelia burgdorferi and Babesia spp.: Clinical Presentation and Diagnosis

*Babesia* spp. can cause human babesiosis, which may present with a range of clinical symptoms depending on the level of parasitemia, patient age, immune status, and the presence of comorbidities. Mild disease may be asymptomatic, whereas moderate disease can present with flu-like symptoms such as fever, headache, myalgia, and arthralgia. In severe cases, babesiosis may lead to hemolytic anemia, acute respiratory distress syndrome, acute kidney injury, bilirubinuria, and even death [20,42,43].

Following a tick bite, *Babesia* spp. enter the capillary circulation, invade red blood cells, then mature and multiply. Once they reach the merozoite stage, they exit and lyse the red blood cells, then invade new erythrocytes [42].

Co-infection with *Borrelia burgdorferi* and *Babesia* spp. has been documented in endemic regions where the two pathogens share the same vector. In a study conducted by Krause et al. [44], 240 patients with Lyme borreliosis were followed longitudinally. Among them, 26 patients (11%) showed evidence of co-infection with *Babesia microti*. These co-infected individuals experienced a more severe clinical course, characterized by prominent symptoms such as headache, fever, splenomegaly, and profuse sweating. Moreover, half of the co-infected patients had symptoms lasting 3 months or longer, compared with only 7 of 184 patients with Lyme disease alone. In another study from Spain, Folgueras et al. [45] followed 120 patients with confirmed Lyme disease, 47 of whom (39.2%) were seropositive for *Ba. venatorum/divergans*. They found that patients with coinfection more frequently experienced cardiorespiratory symptoms, such as dyspnea and atrioventricular (AV) block. Hoversten et al. [46] presented a clinical case of a woman who, at the end of a 21-day course of amoxicillin for Lyme disease, developed a fever of 39.4 °C and myalgia, nausea, and fatigue. Laboratory results revealed anemia and thrombocytopenia. A PCR test for *Ba. microti* DNA was positive, and she was treated with azithromycin and atovaquone for 10 days, which led to an improvement in her general condition, normalization of body temperature, and an increase in erythrocyte, hemoglobin, and platelet counts.

In an experimental study with mice, Djokic et al. [47] found that *Ba. microti* can suppress the acquired immune response, significantly reducing the number of B and T cells in the spleen of mice co-infected with *Bo. burgdorferi* and *Ba. microti* compared to naïve mice. The ELISA method was used to assess the humoral immune response to *Bo. burgdorferi* and showed a suppressed humoral response in co-infected mice. This leads to higher tissue colonization and more severe clinical manifestations of Lyme disease. Destruction of the marginal zone and atrophy of B cells in the spleen begin in the acute phase of parasitemia, resulting in weaker antibody production, which is necessary for clearing *Bo. burgdorferi* from the body. This is observed in younger mice, in which it is assumed that, during co-infection, a Th1-mediated immune response predominates, in contrast to co-infected older mice, in which a Th2-mediated response predominates [48]. It has been found that, in co-infection, inflammatory arthritis is more severe and can persist for up to 16 weeks after infection [49].

On the other hand, infection with *Bo. burgdorferi* activates the TLR2 signaling pathway, leading to activation of macrophages and polymorphonuclear cells. This results in a reduction in parasitemia in mice co-infected with *Ba. microti* and *Bo. burgdorferi* [50,51,52]. Other studies in humans [53] and mouse models [54] have not found a statistically significant difference in the clinical course between infection with *Bo. burgdorferi* alone and co-infection with *Bo. burgdorferi* and *Babesia* spp.

Diagnosis in patients co-infected with *Bbslc* and *Babesia* spp. can be challenging, as both diseases may present with a wide range of clinical signs and overlapping symptoms [55]. As mentioned above, human babesiosis may follow a mild course with flu-like symptoms such as myalgia, fever, and headache. Although rare, LD may present in a similar way, particularly when the initial erythema migrans goes unnoticed. In a study from Italy involving residents of an LD-endemic region, approximately 17% of individuals with confirmed LD reported flu-like complaints [56]. In a meta-analysis by Boyer [57], about 48.1% of patients diagnosed with LD had flu-like symptoms and positive serology or PCR results. According to the recommendations of the Infectious Diseases Society of America (IDSA) [58,59], the diagnosis of LD is based on positive two-tier serology—an initial positive ELISA followed by a positive Western blot—whereas the diagnosis of human babesiosis is based on a positive polymerase chain reaction (PCR) result or visualization of protozoa on a blood smear, which is considered the gold standard [20]. In cases of co-infection with both pathogens, immune dysregulation may occur, potentially leading to negative serological results for *Bbslc* infection and, consequently, delayed diagnosis and treatment [42]. Co-infection with *Bbslc* and *Babesia* spp. should be considered in patients who present with more severe and/or persistent symptoms despite adequate treatment, or with hematological abnormalities such as hemolytic anemia, thrombocytopenia, hepatomegaly, and splenomegaly. In these cases, in addition to serological testing for *Bbslc* infection, PCR testing for *Babesia* spp. and a blood smear are recommended [42].

### 3.2. Co-Infection with Borrelia burgdorferi and Anaplasmosis phagocytophilum: Clinical Presentation and Diagnosis

*An. phagocytophilum* is an obligate intracellular, Gram-negative rickettsia. Unlike other Gram-negative organisms, it lacks surface lipopolysaccharides, which, together with its ability to block lysosome fusion, protects it from neutrophil activity and allows it to survive within these cells. During infection with *An. phagocytophilum*, proinflammatory cytokines are produced, leading to neutrophil degranulation and tissue damage [60].

Similarly to infection with *Babesia* spp., the clinical presentation can range from asymptomatic to severe or, very rarely, fatal. The most common symptoms are nonspecific fever, headache, myalgia, and gastrointestinal manifestations, most frequently diarrhea [20,61].

In a study conducted in Romania involving 80 participants with Lyme disease and a history of tick bites, 10% were seropositive for *An. phagocytophilum* [62]. In another study from Poland that examined the antibody response to *Bo. burgdorferi* infection in 93 forest workers (a high-risk group for tick bites), 28 showed positive serological evidence of co-infection with *Bo. burgdorferi* and *An. phagocytophilum* [63]. In a large Czech study of 314 patients, approximately 8% were seropositive for co-infection with the two pathogens [64]. According to Boyer’s systematic review, the association between erythema migrans and infection with *An. phagocytophilum* is the second most frequently reported [57].

Available clinical studies on co-infection with *Bo. burgdorferi* and *An. phagocytophilum* show only limited and inconclusive evidence that co-infection significantly changes the clinical course of Lyme disease [65,66]. The largest prospective study in early Lyme disease (erythema migrans) found co-infection rates of 2–10%, with a statistically significant increase in symptom number only in a small subgroup of co-infected patients [67]. Overall, disease course, severity, and response to therapy are generally similar to Lyme disease alone [58]. Systematic reviews support that most patients with *Bo. burgdorferi* and *An. phagocytophilum* do not experience clearly worse symptoms, though about one-fifth show additional systemic findings such as fever and hematologic abnormalities [57].

Data from animal models indicate that co-infection with *Bo. burgdorferi* and *An. phagocytophilum* can lead to substantial changes both in pathogen distribution and in the host immune response [68]. In the C3H/HeN mouse model, Holden et al. demonstrated that co-infection with *An. phagocytophilum* results in a broader tissue dissemination of *Bo. burgdorferi* compared with monoinfection, without significantly affecting the bacterial load of Anaplasma, suggesting an asymmetric interaction between the two pathogens [69]. At the same time, co-infected animals exhibit a reduced pathogen-specific antibody response against *An. phagocytophilum*, as well as alterations in the cytokine profile, characterized by decreased Th1-mediated activity (IFN-γ, IL-12) and a relative increase in pro-inflammatory mediators such as IL-6. These changes may facilitate the persistence and dissemination of *Bo. burgdorferi* [57,69]. Overall, data from experimental models suggest that co-infection can amplify tissue involvement and the inflammatory manifestations of Lyme disease, including arthritis, through immunomodulation, providing a biologically plausible explanation for the observed clinical variability and diagnostic challenges in humans with tick-borne co-infections [57].

Similarly to human babesiosis, the clinical presentation of HGA is heterogeneous, with flu-like symptoms being the most common. This may result in delayed diagnosis and, consequently, delayed initiation of treatment. Although serological testing is most frequently used to diagnose *An. phagocytophilum* infection, direct visualization on a blood smear and the use of PCR-based methods are more suitable for achieving an accurate diagnosis [70].

### 3.3. Co-Infection with Borrelia burgdorferi and Ehrlichia spp.: Clinical Presentation and Diagnosis

*Ehrlichia* species cause human ehrlichiosis. Several *Ehrlichia* species have been identified to date: *E. chaffeensis* (human monocytic ehrlichiosis, HME), *E. ewingii* (human granulocytic ehrlichiosis, HGE), *Ehrlichia muris*-like agent (EMLA), and *E. muris*. Similarly to the tick-borne diseases discussed above, the symptoms of ehrlichiosis are nonspecific—myalgia, fever, headache, and sometimes gastrointestinal manifestations or rash [71]. The severity of the clinical presentation varies and depends on the individual’s immunological status, which is why hospitalizations among adult patients are more frequent [72].

Unlike the pathogens discussed so far, we did not find any retrospective or prospective studies comparing patients with Lyme disease alone and those co-infected with *Bo. burgdorferi* and *Ehrlichia* spp. We identified several clinical case reports describing patients who were seropositive for both pathogens, but no significant worsening of the clinical course was reported [57,73,74,75]. Although it is biologically plausible for both pathogens to be transmitted simultaneously by a single tick bite, there are no published data on the simultaneous occurrence or exacerbation of symptoms of both infections.

In experimental mouse models, it has been reported that simultaneous exposure to both pathogens results in significantly more pronounced tissue and joint inflammation, as well as more frequent cytopenic conditions, compared with monoinfected mice. These data support the possibility of an immunomodulatory effect of concurrent infection with *Bo. burgdorferi* and *Ehrlichia* spp. [76].

Serological testing and PCR-based methods are most frequently used to diagnose *Ehrlichia* spp. Infections [71,72].

### 3.4. Co-Infection with Borrelia burgdorferi and N. mikurensis: Clinical Presentation and Diagnosis

Neoehrlichiosis, whose etiological agent is *N. mikurensis* [77], is a relatively new infectious disease, with the first case published in 2014 [78].

*N. mikurensis* is an intracellular, Gram-negative pathogen that infects, survives, and develops in the endothelial cells of blood vessels. Typically, the infection is accompanied by high fever, chills, sweating, thromboembolic vascular events, deep vein thrombosis, and localized muscle pain in the affected blood vessels [77,79].

In a Norwegian study, 10% of samples from patients with confirmed Lyme disease were also positive for *N. mikurensis* [80].

We were unable to find published data on experimental animal models that examined changes in immune response and symptoms in relation to monoinfection with *Bbslc* or *N. mikurensis*.

There are numerous European studies that have reported the simultaneous presence of both pathogens (*Bo. burgdorferi* and *N. mikurensis*) in populations of *Ixodes* ticks [81,82]. The most likely reason for the limited data on co-infection between these pathogens is that *N. mikurensis* is a recently identified pathogen and that PCR-based methods are required for its diagnosis.

### 3.5. Co-Infection with Borrelia burgdorferi and Bartonella spp.: Clinical Presentation and Diagnosis

*Bartonella* spp. are Gram-negative, facultative intracellular pathogens that can cause multisystemic disease, affecting the liver, spleen, lymph nodes, and central nervous system (CNS). *Bartonella* spp. can cause cat scratch disease (*B. henselae*), trench fever (*B. quintana*), Carrion’s disease (*B. bacilliformis*), endocarditis, and aseptic meningitis. The clinical manifestations are diverse and may include fever, neurological symptoms, lymphadenopathy, and others [83,84].

There are ample data in the literature demonstrating the presence of *Bartonella* spp. DNA in *Ixodes* ticks [85,86,87], but there is still no conclusive evidence that humans can be infected via tick bite [88,89].

Cases have been reported of patients with Lyme disease in whom infection with *Bartonella* spp. has been demonstrated serologically or by PCR [90]. Two of these involved patients with symptoms characteristic of neuroborreliosis who were followed over time [91]. In the study by Eskow et al., four patients with headache, fever, insomnia, and depression all tested positive for *B. henselae* infection, but only three had *Bbslc* DNA detected in their cerebrospinal fluid (CSF) [91]. In another study from Poland, 2 of 17 patients with neuroborreliosis tested positive for *B. henselae* DNA in CSF [92]. However, there are no controlled studies in the literature comparing the course of simultaneous infection with *Bbslc* and *Bartonella* spp. with monoinfection by either pathogen.

Despite evidence of serological and ecological co-exposure between *Bo. burgdorferi* and *Bartonella* spp. [85,86,87,90,91,92], we were unable to find published data on animal models in which clinical and immunological outcomes of simultaneous infection with both pathogens were monitored.

The diagnosis of *Bartonella* spp. is complex and involves several methods: serological testing for IgM/IgG titers, PCR detection, culture isolation of the pathogen, and histopathology. Each of these methods has limitations, but the most conclusive are histopathology (which is also the most invasive for the patient) and culture isolation of the pathogen (which delays diagnosis due to slow bacterial growth). For the most accurate diagnosis, at least two of these methods should be used in combination [93].

### 3.6. Co-Infection with Borrelia burgdorferi and Rickettsia spp.: Clinical Presentation and Diagnosis

*Rickettsia* spp. are Gram-negative intracellular pathogens transmitted by arthropods that cause numerous diseases in humans [94].

Epidemiological studies of ticks have found simultaneous carriage of *Bbslc* and *Rickettsia* spp., including *R. helvetica* (which causes Aneruptive fever) and *R. monacensis* (which causes Mediterranean spotted fever-like disease) [95,96,97]. This suggests that simultaneous transmission of both infections to humans is possible. However, there are no large studies in the literature on the effect of these two pathogens on the clinical presentation. In a study by Tijsse-Klasen et al., out of 67 skin biopsies examined, 47 were positive for Bbslc and only one of these was also positive for *R. monacensis*, but the symptoms were consistent with Lyme disease, so the effect of *Rickettsia* spp. was not documented [97]. We were unable to find any animal model studies examining co-infection with *Bbslc* and *Rickettsia* spp.

Acute rickettsioses usually present with a characteristic clinical picture, and the differential diagnosis between LD and rickettsioses should not be difficult [98]. However, in the context of more acute symptoms, the diagnosis of LD may be overlooked. *Rickettsia* spp. infections are confirmed based on clinical presentation and serological and molecular techniques.

### 3.7. Co-Infection with Borrelia burgdorferi and Borrelia miyamotoi: Clinical Presentation and Diagnosis

The infection with *Bo. miyamotoi* has a biphasic course, with the first episode dominated by flu-like symptoms, fever, headache, arthralgia, and myalgia, lasting 4–5 days, followed by a relapse of symptoms approximately 9 days later [99,100]. Cases involving the central nervous system have been reported [101,102].

A Dutch study found that 7.4% of patients with confirmed Lyme neuroborreliosis were also positive for *Bo. miyamotoi* infection [103]. Together with the proven simultaneous presence of both pathogens in *Ixodes* ticks [104,105], this finding demonstrates that Bo. miyamotoi can be a co-infection in LB.

However, we did not identify any large clinical studies in humans or experimental animal models that specifically examined co-infection with these two pathogens and how it alters the clinical presentation or immunopathology.

A Russian study comparing patients with LB and *Bo. miyamotoi* infection showed that patients with relapsing fever seek hospital care earlier, as the acute stage of *Bo. miyamotoi* infection progresses more rapidly than in LB [11].

*Bo. miyamotoi* infection is detected using serological and molecular methods; a blood smear can also be used [100].

### 3.8. Co-Infection with Borrelia burgdorferi and Tick-Borne Encephalitis Virus: Clinical Presentation and Diagnosis

The tick-borne encephalitis virus (TBEV) is a spherical, enveloped RNA virus belonging to the genus *Flavivirus* in the family *Flaviviridae* [106]. It is found mainly in Northern and Central Europe as well as in Asia [106]. TBEV has pronounced neurotropism and causes a typically biphasic illness. The first phase is characterized by flu-like symptoms, while the second phase may present with signs of meningitis and/or encephalitis—such as headache, paralysis, and confusion. However, the second phase may not develop, and in some cases the infection remains asymptomatic [21].

In a study from Latvia, 51 patients with confirmed dual infection with *Borrelia burgdorferi* and TBEV were followed. These patients showed a higher frequency of neurological symptoms, biphasic fever, and overlapping systemic manifestations characteristic of both diseases [107]. In a systematic review summarizing data from 655 patients, the most common serologically proven co-infection was between *Bbslc* and TBEV, which was also among the most frequently confirmed active co-infections [57].

In a mouse study, Porcelli et al. [108] observed that the clinical expression of co-infection depends on the timing of pathogen exposure. Mice infected first with *Bo. afzelii* and then with TBEV 9 days later developed more severe symptoms and had a higher viral load than those infected with TBEV 21 days after *Bo. afzelii* infection. Simultaneous infection led to a more moderate clinical course and no deaths. These findings highlight the complexity of the underlying immunological mechanisms and the ways in which the two pathogens influence each other.

Because the two conditions share many clinical features and have similar incubation periods, distinguishing Lyme disease from *TBEV* infection can be challenging. This is particularly true in cases of co-infection by both pathogens, where their symptoms overlap. A possible co-infection should be suspected when there is an epidemiological connection between the two diseases, unusually severe neurological manifestations, and a biphasic fever pattern. Confirmation of *TBEV* infection relies on serological testing and molecular techniques such as PCR. The same diagnostic principles apply to the other viral infections discussed (*Powassan*, *Louping ill*, and *Eyach*).

### 3.9. Co-Infection with Borrelia burgdorferi and Other Tick-Borne Viruses: Clinical Presentation and Diagnosis

As mentioned above, *Ixodes* ticks can also transmit *Powassan*, *Louping ill*, and *Eyach viruses* [15,16,17].

*Powassan virus* is a *flavivirus*, and the disease is rare, occurring in the United States and Canada. The clinical presentation ranges from asymptomatic infection to encephalitis, with symptoms such as fever, headache, vomiting, confusion, paralysis, and seizures [109].

A study from Wisconsin found that 16.4% of 55 Lyme disease–positive samples were also positive for *Powassan virus* [110]. However, there are insufficient controlled studies in the literature to compare differences in symptoms between mono-infection and co-infection with Powassan virus.

*Louping ill* virus, similar to *Powassan virus* and *TBEV*, belongs to the genus *Flavivirus* and can likewise cause a wide range of clinical manifestations, from asymptomatic infection to encephalitis [111]. *Eyach virus* belongs to the genus *Coltivirus*, like *Colorado tick fever virus*, and is transmitted by *I. ricinus* in Europe, with endemic areas in Germany and France [18]. The disease is rare in humans and, like the other viruses discussed, can present with diverse clinical features, ranging from asymptomatic or flu-like illness to neurological involvement such as meningitis, encephalitis, or polyneuritis [112].

We were unable to find experimental animal models that examined co-infection between the viral pathogens considered and Bbslc, which tracked changes in the clinical presentation and immunopathology during simultaneous infection with these pathogens.

## 4. Treatment and Prevention of Lyme Disease and Co-Infections

**Figure 2:**
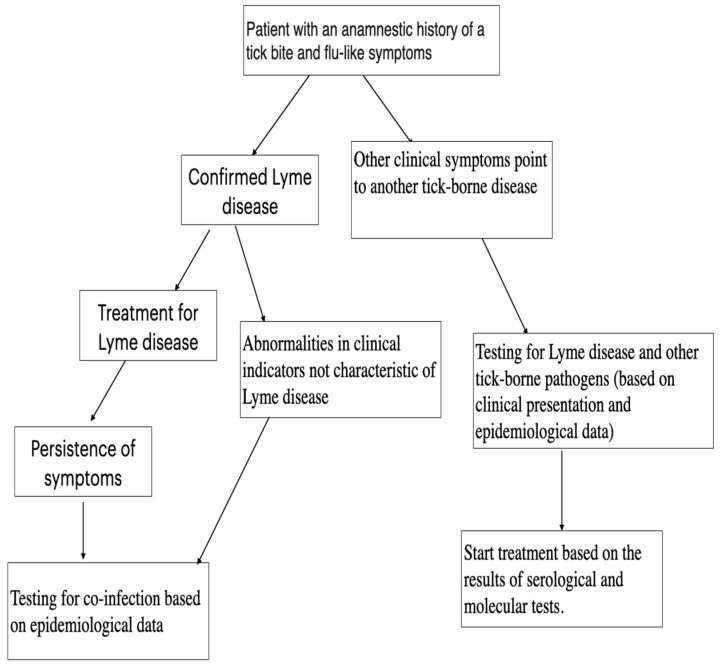
Diagnostic approach in patients with a history of tick bites.

Accurate diagnosis is essential for appropriate treatment. The diagram below presents, see Figure 2 a diagnostic algorithm that can be used when co-infection in Lyme disease is suspected.

Standard therapy for LB includes doxycycline, ceftriaxone, or amoxicillin, which are also effective against *Rickettsia* spp., *An. phagocytophilum*, *Ehrlichia* spp., and *N. ehrlichia* [58]. However, this regimen does not cover *Babesia* spp. or viral infections. Management of viral infections requires supportive, pathogenetic treatment. Babesiosis necessitates the use of antiparasitic agents: in mild cases, a combination of atovaquone and azithromycin is preferred, whereas in more severe cases, a combination of clindamycin and quinine may be used [20,59]; see Table 2.

To optimize the choice of antimicrobial therapy, randomized clinical trials are needed to evaluate the effects of different antimicrobial combinations in various co-infections. In addition, both in vitro and in vivo studies are required.

**Table 2:** Most common antibiotics and their combinations for the treatment of tick-borne pathogens.

*Borreli burgdorferi* s.l. Complex	*Borrelia miyamotoi*	*Babesia* spp.	*An. phagocytophilum*	*Ehrlichia* spp.	*N.* *mikurensis*	*Rikettsia* spp.	*Bartonella* spp.	Tick-Borne Virus
doxycycline [58]		atovaquone and azithromycin [20,59]	doxycycline [58,83]					supportive treatment [18,106,110]
ceftriaxone [58]		clindamycin and quinine [20,59]	rifampin [21]			azithromycin [21,83]		
Amoxicillin [58]						fluoroquinoles [21,83]		

Abbreviations: s.l.—sensu lato; spp.—multiple species.

Prevention of tick-borne diseases remains one of the most important measures for addressing this public health problem. There are various approaches to tackling it, including controlling tick populations through ecological, biological, and chemical methods [7]. The literature also describes the development of anti-tick vaccines that can prevent pathogen transmission [7,113],. Another strategy is the use of human monoclonal antibodies, which in experimental mouse models have shown both protective and therapeutic effects against TBEV [114] and Powassan virus [115]. Similar results have been obtained by Schaible et al. [116], who demonstrated that passive immunization of mouse models with human monoclonal antibodies against OspA protects against Bb infection.

TBEV is one of the few tick-borne pathogens for which there are licensed vaccines with proven efficacy. There are still no approved vaccines against Lyme disease, but several candidates are currently in clinical trials [7].

## 5. Conclusions

*Ixodes* ticks are the primary vectors of Lyme disease, but they can carry multiple pathogens, meaning that their bites carry the risk of transmitting more than one pathogen. These pathogens have overlapping clinical presentations, add new symptoms, or prolong the course of Lyme disease. For some of the pathogens we present (*Rickettsia* spp. and *Bo. miyamotoi*), the acute phase is more severe, which can delay the diagnosis of Lyme disease. Viral infections affecting the CNS are associated with more frequent and more severe neurological complications, while other co-pathogens (*Babesia* spp.) may prolong and exacerbate the symptoms of Lyme disease (LD). Despite the data presented in the literature, there are still no unified protocols for the diagnosis and treatment of co-infections in Lyme disease, and large clinical and experimental studies are needed to assess the burden of these diseases, as well as the implementation of new methods or combinations of diagnostic approaches for their detection. Patients with Lyme disease should be asked in detail about where and when they may have encountered an *Ixodes* tick, and clinicians should be familiar with the geographical distribution of Ixodes-borne diseases. Newer molecular methods for pathogen detection should be implemented in clinical practice, and clinicians should be aware of the characteristic clinical and laboratory abnormalities that may indicate co-exposure to more than one pathogen

## Figures and Tables

**Table 1:** Examples of co-infections in Ixodes ricinus ticks in Europe and their relevance to Lyme disease co-infections.

No	Author (Year)	Country/ Region	Pathogens Studied	% ≥1 Pathogen	% ≥2 Pathogen	Most Common Combinations
1	Oechslin et al. (2017) [22]	Switzerland	*Borrelia s.l.*, *B. miyamotoi*, *Anaplasma*, *Neoehrlichia mikurensis*, *Rickettsia* spp., *Babesia* spp.	30.9%	20.0%	*Borrelia s.l.* + *Rickettsia*, *Borrelia* + *Anaplasma*
2	Lommano et al. (2012) [23]	Switzerland	*Borrelia s.l.*, *Anaplasma*, *Babesia*, *Rickettsia*	31%	13%	*Borrelia* + *Anaplasma*
3	Pawełczyk et al. (2021) [24] *	Poland	*Borrelia s.l.*, *Babesia* spp.	10–24%	1.5–5%	*Borrelia* + *Babesia microti*
4	Jaenson et al. (2024) [26]	Northern Europe	*Borrelia s.l.*, *Babesia* spp.	~20%	2–4%	*Borrelia* + *Babesia venatorum*
5	Olsthoorn et al. (2021) [29]	Western Europe	*Borrelia s.l.*, *Anaplasma*, *Neoehrlichia mikurensis*, *Rickettsia* spp., *Babesia* spp.	25–30%	8–12%	*Borrelia* + *Anaplasma*, *Borrelia* + *Neoehrlichia*
6	Sawczyn- Domańska et al. (2023) [25]	Poland	*Borrelia s.l.*, *B. miyamotoi*, *Neoehrlichia mikurensis*, *Babesia* spp.	28.6%	9.4%	*Borrelia* + *Babesia*, *Borrelia* + *Neoehrlichia*
8	Sprong et al. (2018) [28]	Europe	Review	–	–	*Borrelia* + *Anaplasma* + *Babesia*
8	Nader et al. (2018) [27] *	Bulgaria	*Borrelia s.l.*, *Anaplasma*, *Neoehrlichia mikurensis*, *Rickettsia* spp., *Babesia* spp.	23.2%	2.5% (mainly in *Ixodes* spp.)	*Babesia* spp. + *other pathogens* *Rikettsia* spp. + *Borrelia s.l.*

Abbreviations: s.l.—sensu lato; spp.—multiple species. *—Various types of ticks have been studied.

## Data Availability

No new data were created or analyzed in this study. Data sharing is not applicable to this article.
